# Reduction in pregnancies and litters in mice couples with splenectomized male

**DOI:** 10.1590/ACB360201

**Published:** 2021-02-22

**Authors:** Dalton Muniz Santos, Gisele Araújo Pereira, Kelly Renata Sabino, Andy Petroianu

**Affiliations:** 1 MD, MSc. Universidade Federal de Minas Gerais – School of Medicine – Department of Surgery – Belo Horizonte (MG), Brazil.; 2 MD. Universidade Federal de Minas Gerais – School of Medicine – Department of Surgery – Belo Horizonte (MG), Brazil.; 3 PhD. Universidade Federal de Minas Gerais – School of Medicine – Department of Surgery – Belo Horizonte (MG), Brazil.; 4 PhD, Full Professor. Universidade Federal de Minas Gerais – School of Medicine – Department of Surgery – Belo Horizonte (MG), Brazil.

**Keywords:** Spleen, Splenectomy, Reproduction, Sexual disfunction, Fertility

## Abstract

**Purpose:**

The spleen is relevant in blood purification, hematopoiesis, metabolism, and immune response to antigens, in addition to the storage and control on the release of metals and amino acids. Its functions concerning reproduction characteristics are still unknown. The objective was to study the influence of splenectomies on reproduction.

**Methods:**

This study analyzed 25 mice couples, distributed into five groups: group 1 – control, no surgery: group 2 – control, submitted to laparotomy and laparorrhaphy only; group 3 – splenectomy in male mice; group 4 – splenectomy in female mice; group 5 – splenectomy in male and female mice. The animals were studied as regards the number of gestations and offspring generated in each gestation.

**Results:**

A decrease in both the number of gestations and the number of offspring was verified in the male mice that had received a splenectomy when coupled with normal female mice. It is important to emphasize lower reproduction level when paired asplenic males with normal females, otherwise, the couples in which both mice had been splenectomized did not present change in the reproduction pattern.

**Conclusions:**

A reduction in the number of pregnancies and litters occurs in mice couples when the male mice were previously splenectomized.

## Introduction

The spleen performs important functions of defense, such as the removal of antigens from the blood flow, the storage of macrophages, as well as the production of lymphocytes, monocytes, and opsonins. The splenectomy is the main treatment for the majority of diseases of this organ. The asplenic state is related to immunodeficiency and may provoke fatal sepsis. Asplenia is also associated with hematological alterations, reduction in release of mature leukocytes to combat sepsis, increasing in number of platelets and metabolic alterations, such as increase in fatty infiltration of the liver, increase in weight, rise in triglyceride and cholesterol levels^1^^,^ 2. Changes in eating habits, and organic defense, with deficiency in the removal of bacteria, fungi, viruses, and foreign bodies from the blood flow, increasing the susceptibility of the splenectomized mice to infection were also described^1^^–^ 7.

During the Battle of Dettingen, in England in 1743, a soldier had his spleen removed due to an open wound on that organ. After the splenectomy, the soldier reported loss of interest in sexual relations without any other occurrence, which could be related to this change in his will. In the nineteenth century, Shulz and Czermak^8^ observed a decrease in the fertility of splenectomized animals. These facts raised the hypothesis of splenectomy interfering in sexual activities caused by psychogenic or, more probably, metabolic factors related to the asplenic status.

Saito *et al*.^7^ verified that the splenectomy caused delayed ovulation in rats, which was normalized after receiving an injection of splenocytes. According to these authors, the spleen shapes the ovarian function. The decrease of prolactin in the final phase of a pseudogestation in splenectomized mice suggests that the splenocytes are involved in the mechanism of luteolysis^9^.

Oakley *et al*.^10^ studied ovulation in the presence of inflammatory reactions and verified the rupture of the follicle and the expulsion of the ovulum. There appears to be an inverse relation between the number of leukocytes in the ovary and ovulation, with a decrease in the number of leukocytes infiltrated in the ovaries of splenectomized mice, suggesting that the spleen releases leukocytes during the periovulatory period^11^^–^ 14. Oophorectomy is also related to the metabolism of the glucose and lipids leading to experimental diabetes mellitus and hypercholesterolemia^16^. Otherwise, diabetes mellitus is related to men lower fertility mainly in more advanced age^15^^,^ 16. Although there are indications of the relation between the spleen and reproduction, no studies have been published that associate the spleen with reproduction. The present work aimed to verify the influence of splenectomy on the reproduction process using the animal model.

## Methods

This work belongs to a line of research and was approved by the Ethical Committee in Experimental Research from the Universidade Federal de Minas Gerais (UFMG), under the protocol number 095/11 and strictly followed the criteria set forth in Resolution 879/08 from the Federal Board of Veterinarian Medicine and by Brazilian Federal Law 11.794, which regulates the use of laboratory animals and followed the *Animal Research Reporting of in Vivo Experimental* (ARRIVE) guideline.

This study used 50 adult albino mice (*Mus musculus*) of the BALB/c breed, of which 25 were males and 25 females, with an average weight of 30 g. These animals were kept in separate cages, with two animals, one male and one female, in each cage. They were maintained in a room temperature environment, with natural lighting, receiving rations and water *ad libitum*, and were taken care of daily. The cages were cleaned, and the bedding was changed each day.

All of the couples of mice were followed up over a 60-day-period, during which time the mice were expected to have two consecutive litters. This procedure confirmed the fertility of the couples and revealed the number of offspring per litter per couple. Next, the 50 mice were randomly distributed into five groups (n = 10):

- Group 1: Control, no surgery;
- Group 2: Laparotomy e laparorrhaphy, without any intra-abdominal procedure;
- Group 3: Total splenectomy only in the male mice;
- Group 4: Total splenectomy only in the female mice;
- Group 5: Total splenectomy in both the male and female mice.

The surgical procedures were conducted under general anesthesia using pentobarbital, at a dose of 3.5 mg/animal (90 mg/kg) and fentanyl citrate (2.5 μg/animal), both by intraperitoneal injection. The anesthetized mice were placed in a dorsal position. After the hair removal of the entire abdomen, antisepsis of the trunk of the body was performed using a 2% iodine alcohol solution. Sterilized tissue fields were used to protect the area to be operated on.

In all groups, except group 1 (control), a median laparotomy of 2 cm in length was performed on the upper part of the abdomen. In group 2, the surgical procedure was limited to the laparotomy and laparorrhaphy, without any procedure on the spleen. In the animals from groups 3, 4 and 5, the spleen was identified and its vessels were ligated with a 5-0 silk thread and cut. Next, the spleen was removed. The abdominal cavity of the mice was closed with two continuous sutures (muscles and skin), using 4-0 nylon thread.

Each animal was examined daily, searching for complications and behavioral changes. The reproduction time and the number of offspring for each couple was recorded. This study considered only the couples that reproduced two consecutive times. The animals that did not reproduce in the first part of this study were excluded and substituted by other mice with the same characteristics and that had produced two consecutive reproductions.

In group 1, the study time consisted of the period corresponding to the two pregnancies (42 days). By contrast, in the other groups (2, 3, 4, and 5), the time was of 66 days after surgery, corresponding to three gestations.

The first gestation was not considered, given that the fertilization may have occurred by another male before the beginning of this study. However, the data obtained from the second and third pregnancies were included in this work. The couples that did not reproduce a third time within the two postoperatory months were considered nonreproductive, though they were previously fertile.

The data were presented as mean and standard error of the mean. The results obtained from the reproduction pattern were analyzed by the Fisher’s exact test, used for small samples, which allows one to calculate the probability of the association of independent characteristics. To compare the number of offspring, reproduction time, and hormonal doses, the Kolmogorov–Smirnov normality test was applied, followed by the analysis of variance (ANOVA) and Tukey’s multiple comparison tests. All results were considered to be significant when the differences were correspondent to a probability of higher than 95% (p < 0.05).

## Results

During the entire period of the experiment, the mice were healthy and adapted well the vivarium environment. No complication was verified in any animal during or after the surgical procedures. The mice recovered spontaneously from the surgery and uneventfully returned to their usual activities.

In group 3, three of the five couples with splenectomized males did not reproduce (p = 0.038), indicating an influence in the reproduction pattern, as shown in Table 1. However, the decrease in the number of offspring from breeding of this group was not significant (p = 0.11) (Table 1). On the other hand, no change in the number of offspring could be observed when both male and female mice from the couple were splenectomized.

**Table 1:** Pregnancy and litter obtained from the previously fertile mice couples.

Groups		Pregnancies				Litter		p		
		Yes		No		Average		Gestation		Litter
Couple of micewith no surgery		5		0		7		-		-
Laparotomyon bothmice withoutsplenectomy		5		0		7		1.000		1.00
Splenectomizedmale andnormal female		2		3		2		0.038		0.11
Splenectomizedfemale and normal male		5		0		6		1.000		0.33
Splenectomizedmale and female		5		0		5		1.000		0.27

All groups were compared (ANOVA) with couple of mice with no surgery.

## Discussion

It is important to emphasize lower reproduction level when paired asplenic males with normal females, otherwise the couples in which both mice had been splenectomized did not present change in the reproduction pattern. This result may be due to the interference of the asplenic state in the reproduction process of male mice or rejection between asplenic males and normal females. Anyway, the male asplenic mice demonstrated the same reproductive capacity as the normal mice when coupled with asplenic female mice. Considering that usually asplenic men are married with normal women, this experimental finding may be relevant^17^^–^ 23.

It seems to be pivotal to proceed in the same line of work to clarify the role of the spleen and the repercussions of the asplenic state on reproductive, hormonal, and behavioral functions^24^. Experimental studies in rats have shown that aging interferes in spermatogenesis^25^. Findings from a study conducted by Oakley *et al*.^10^ indicate the interference of the splenectomy on ovulation; however, these findings were restricted to the alterations in female mice only. These results differ from the present study in which no changes in the fertility of the female mice were observed.

This study referred only to male fertility; however, the influence of the splenectomy may occur not only on male fertility, but also on other conditions, such as hypoandrogenism followed by poor healing wounds, physical capacity and other metabolic disorders^2^^,^ 26 ^,^ 27.

The findings similar to this work have not been published in prior literature, and no clarifications were found to explain the noninterference in the group in which both the male and female mice were splenectomized. This result may well be related to the disinterest in sexual activity after splenectomy, as was reported in the eighteenth and nineteenth centuries. Further studies must be carried out in an attempt to allocate greater funding for the comprehension of the splenic function in sexual activities, ovulation, and spermatogenesis.

## Conclusion

A decrease in the number of pregnancies and litters occurs in couples of mice when male mice was previously splenectomized.
